# Transfemoral hepatic vein access in double vein embolization – initial experience and feasibility

**DOI:** 10.1186/s42155-024-00478-y

**Published:** 2024-09-03

**Authors:** Ulrik Carling, Sigurd Berger, Eyvind Gjønnæss, Bård Røsok, Sheraz Yaqub, Kristoffer Lassen, Åsmund Avdem Fretland, Eric Dorenberg

**Affiliations:** 1 Department of Radiology, Rikshospitalet, Oslo University Hospital, Postbox 4950 Nydalen, Oslo, 0424 Norway; 2 Department of Hepato-Pancreatic-Biliary Surgery, Rikshospitalet, Oslo University Hospital, Oslo, Norway; 3 Institute of Clinical Medicine, University of Oslo, Oslo, Norway; 4 Institute of Clinical Medicine, UiT, The Arctic Univeristy of Norway, Tromsø, Norway; 5 The Intervention Centre, Oslo University Hospital, Oslo, Norway

**Keywords:** Double vein embolization, Liver augmentation, Liver hyperthrophy, Portal vein embolization, Post hepatectomy liver failure

## Abstract

**Background:**

Hepatic vein embolization in double vein embolization (DVE) can be performed with transhepatic, transjugular or transfemoral access. This study evaluates the feasibility and technical success of using a transfemoral access for the hepatic vein embolization in patients undergoing preoperative to induce hypertrophy of the future liver remnant (FLR).

**Material and methods:**

Retrospective analysis of single center cohort including 17 consecutive patients. The baseline standardized FLR was 18.2% (range 14.7–24.9). Portal vein embolization was performed with vascular plugs and glue through an ipsilateral transhepatic access. Hepatic vein embolization was performed using vascular plugs. Access for the hepatic vein was either transhepatic, transjugular or transfemoral. Technical success, number of hepatic veins embolized and complications were registered. In addition, volumetric data including degree of hypertrophy (DH) and kinetic growth rate (KGR), and resection data were registered.

R: Seven of the 17 patients had transfemoral hepatic vein embolization, with 100% technical success. No severe complications were registered. In the whole cohort, the median number of hepatic veins embolized was 2 (1–6). DH was 8.6% (3.0–19.4) and KGR was 3.6%/week (1.4–7.4), without significant differences between the patients having transfemoral versus transhepatic /transjugular access (*p* = 0.48 and 0.54 respectively). Time from DVE to surgery was median 4.8 weeks (2.6–33.9) for the whole cohort, with one patient declining surgery, two having explorative laparotomy and one patient having change of surgical strategy due to insufficient growth.

**Conclusion:**

Transfemoral access is a feasible option with a high degree of technical success for hepatic vein embolization in patients with small future liver remnants needing DVE.

## Background

Before large liver resections, liver augmentation procedures might be necessary to induce volume and function growth of the future liver remnant (FLR), to reduce the risk for post hepatectomy liver failure (PHLF) [16]. Portal vein embolization (PVE) has been the gold standard procedure in this setting, proven to yield reliable FLR hypertrophy with little procedure related morbidity [14, 15]. However, it has also been reported that up to 30% of patients do not reach resection [13], primarily due to tumor progression or failure of FLR growth. In 2012, associated liver partition and portal vein ligation in staged hepatectomy (ALLPS) was described as an effective way of increasing FLR volume, but the method has been associated with a relatively high rate of complications. Hence, a less invasive method for liver augmentation has been argued for [11]. In 2016, the first reports on combining PVE with simultaneous hepatic vein embolization emerged [7]. Over time this technique has proven safe and effective, and results in smaller cohorts have indicated a more pronounced FLR hypertrophy and a higher degree of resectability compared to PVE [9]. Larger randomized controlled trials, such as the HYPER-LIV01 [5] and Dragon 2 (Clinical trials nr NCT05428735), comparing the techniques are ongoing. The combined embolization of the portal and hepatic veins has been named either liver venous deprivation (LVD) or double/dual vein embolization (DVE). LVD combines vascular plugs and N-Butyl Cyanoacrylate (NBCA) glue for the hepatic vein embolization, whereas DVE uses vascular plugs only (sometimes combined with coils). Most reports on DVE/LVD include cases where a transhepatic or transjugular access has been used for the hepatic vein embolization [4, 9]. Although the latest standard of practice document mentions transfemoral access as an alternative for hepatic vein embolization [1], the data is scarce with one publication mentioning one case were this access was used in a patient with multiple accessory veins [3]. Just recently a study on 23 patients comparing transjugular and transfemoral access was published [20], indicating a faster procedure time and non-inferior FLR hypetrophy in the transfemoral group. In our institution, DVE has been included in the liver augmentation armamentarium, and is used primarily for patients with standardized FLR < 20%. Different accesses to the hepatic veins have been used, and the purpose of this study is to report the feasibility of using a transfemoral access for the hepatic vein embolization in DVE.

## Material and methods

This is a retrospective analysis of a prospective single center cohort of 17 consecutive patients that underwent DVE during 2020–2023. Data collection from medical journal and radiological information systems was approved by the local data protection official, with waiver of patient consent.

PVE was performed as previously reported [2]. In short, an ultrasound guided ipsilateral portal vein branch was accessed. A 3D portogram with cone beam CT was performed, and embolization was performed with N-Butyl Cyanoacrylate (NBCA) glue and a central vascular plug (Amplatzer™ Vascular Plug II – AVP II; Abbott Laboratories Chicago,USA). If segment 4 was embolized, microcoils were used to avoid the risk of non-target embolization.

Hepatic vein embolization was done using vascular plugs (AVP II) only, oversized by at least 50%, and typically one plug per vein. The access for hepatic vein embolization was chosen by the operator based on anatomy and the appreciated number of veins needing separate embolization. In patients where a transhepatic access was used, a peripheral hepatic vein branch was punctured and accessed with a 4F introducer (Cordis Corporation, Miami Lakes, USA) before PVE. After the completion of PVE the 4F sheath was exchanged to a 23 cm 7F vascular sheath with a radiopaque tip (Cordis Corporation, Miami Lakes, USA). In two cases an additional 7F cobra-shaped catheter (Boston Scientific, Marlborough, USA) was used for separate embolization of cranial hepatic vein branches. In patients where a transjugular access was used, following the completion of the PVE, the internal jugular vein was punctured and a 7F sheath (Flexor; Cook Medical, Bloomington, USA) was placed in the target hepatic veins using a catheter (4F MPA; Cordis Corporation, Miami Lakes, USA) and wire. In the cases where a transfemoral access was used, the femoral vein was punctured after the completion of PVE and the target liver veins were accessed using an angled catheter (typically a 4-5F cobra shaped catheter) followed by a 7F or 8F sheath (Flexor; Cook Medical, Bloomington, USA). The veins were then embolized using AVP II. Decisions regarding the number of hepatic veins to be embolized in each case were based on the individual anatomy, which was assessed by CT imaging prior to the procedure. All veins involved in drainage of the major part of the liver to be resected were considered for embolization.

Volumetric assessment was performed on either Magnetic Resonance Imaging or CT performed prior to DVE and on contrast enhanced CT 1–3 weeks after the procedure. FLR volume was measured manually as described before [2] and standardized FLR (sFLR) was calculated related to the body surface area [18].

The primary endpoint was technical success defined as placement of plugs in the hepatic veins in the part of the liver to be resected. Also technical aspects such as number of veins embolized and sizes of plugs used were registered. Complications registered in the medical journals were classified as according to the CIRSE classification [6]. Secondary endpoints included volumetric changes and resection rates. Hypertrophy data including degree of hypertrophy (DH) and kinetic growth rate (KGR), defined as sFLR% change and sFLR% change/week [19] were registered. In addition, resection rate and posthepatectomy liver failure (PHLF) [17] was registered.

### Statistics

Due to the limited number of patients, median with range was used for continuous data. For comparisons of groups, Mann–Whitney U-test or Chi-squared test were (IBM SPSS 29.0; Corp., Armonk, USA). A *p*-value < 0.05 was considered as statistically significant. As transjugular and tranhepatic access has been the standard accesses, these were combined into one group and compared to transfemoral access alone.

## Results

**Fig. 1:**
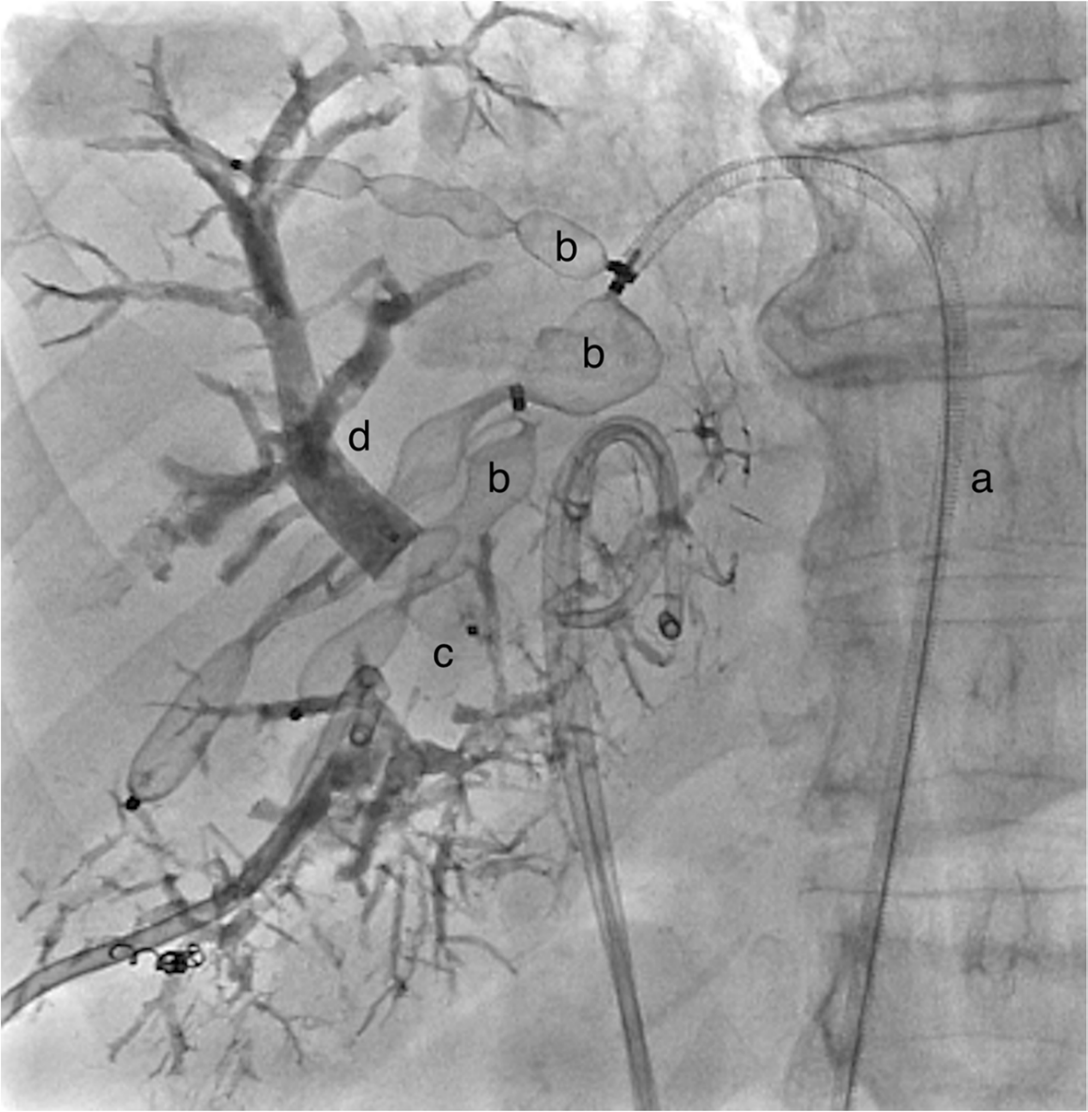
Double vein embolization with transfemoral access for the hepatic vein embolization of multiple veins. A vascular sheath (**a**) and vascular plug (**b**) is placed in the main right heaptic vein. Additional plugs (**b**) separate hepatic vein branches. A vascular plug (**c**) has also been placed in the right portal vein, which is also filled with glue (**d**)

Demographics and pre-embolization data for the cohort is reported in Table 1. In total, of the 17 patients that underwent DVE, 7 had transhepatic, two had transjugular and 7 had transfemoral access for the hepatic vein embolization. The technical success using transfemoral access was 100% compared to 80% for transhepatic (71%) and transjugular (100%) combined. The right hepatic vein was intended to be embolized in all patients but one, in whom the middle vein was the target. Two patients had both their right and middle vein embolized. Furthermore, an accessory right hepatic vein was embolized in 4 patients. A median of two plugs (1–6) were used in each patient for the hepatic vein embolization, one plug per vein, with up to 6 plugs used in one patient with transfemoral access. Figure 1 shows an example of multiple plug embolization through a transfemoral access. AVP II of median size 16 mm (8–22) was used, median 14 mm (8–22) in the transfemoral group and 18 mm (10–22) in the transhepatic/transjugular group (*p* = 0.19). Five complications after DVE were registered, none of them were regarded as severe. Two patients had grade 3 complications of spontaneously resolving abdominal pain. One periprocedural grade 1 complication was recorded in a patient with transjugular access with dissection of an accessory right vein. Despite this, the vein was still successfully embolized. No access site complications were registered in any of the patients. However, one patient with transhepatic access was embolized in the middle vein instead of the right hepatic vein, which was discovered on the follow up CT. This patient with colorectal liver metastases had a change of strategy and was ultimately treated by local resections and ablations. In another transhepatic case, we were not able to selectively embolize a large cranial branch, as we were unsuccessful in placing a large enough catheter in the vein. This patient underwent re-embolization of both portal and hepatic vein branches (transfemorally) after some time due to insufficient growth, and resection was possible after 33 weeks.

**Table 1:** Demographic data of the cohort of patients (*N* = 17) undergoing double vein embolization

Female Gender/%	9/53
Age in years median (range)	68.2 (47–85)
Etiology	
Colorectal liver metastases	13
Peri-hilar cholangiocarcinoma	3
Hepatocellular carcinoma	1
BMI^a^ kg/m^2^ median (range)	24.1 (19.5–32.3)
FLR^b^ volume ml median (range)	285 (219–347)
sFLR^c^ % median (range)	18.2 (14.7–24.9^d^)

^a^*BMI* body mass index

^b^*FLR* future liver remnant

^c^standardized FLR

^d^One patient with only segment 1 and 4 as FLR had double vein embolization despite sFLR 24.9%

The volumetric changes of the FLR assessed on CT after median 2.1 weeks (1–2.9), were not different between the groups, as seen in Table 2. The number of patients reaching surgery was not different between the groups. One patient with transjugular access reaching sufficient FLR volume, ultimately declined surgery. All other patients were resected, except two patients (one in each group) having only explorative laparotomy (one with perihilar cholangiocarcinoma with positive lymph node and one with CRLM metastases with high portal pressure and progression). Ten patients underwent right-sided hemihepatectomy and three extended right-sided, and one patient had as earlier mentioned local resection and ablations. Time to surgery was approximately 5 weeks, see Table 2.

**Table 2:** Results after double vein embolization. Numbers in median (range)

	All	Transfemoral	Transjuglar/transhepatic	*P*-value
Segment 4 portal vein embolization	3	0	3	0.12
Number of hepatic veins	2 (1–6)	2 (2–6)	1.5 (1–3)	0.09
Number of plugs	2 (1–6)	2 (2–6)	1.5 (1–3)	0.09
FLR^a^ post DVE ml	414 (311–547)	414 (338–547)	401.5 (311–526)	0.89
FLR change %	48.7 (15.7–105.6)	51.1 (19–105.6)	42.1 (15.7–81,7)	0.54
sFLR^b^ % at first evaluation	25.8 (19.9–37.7)	25.8 (23.8–37.7)	27 (19.9–35.3)	0.36
DH^c^	8.6 (3.0–19.4)	9.1 (3.8–19.4)	7.65 (3–15.9)	0.48
KGR^d^	3.6 (1.4–7.4)	3.6 (3.2–7.1)	3.6 (1.4–7.4)	0.54
CIRSE grade^e^				
3	2	1	1	
2	2	0	2	
1	1	0	1	
Time to Surgery	4.8 (2.6–33.9)	4.9 (2.6–12.1)	4.7 (2.7–33.9)	0.68
Did not reach surgery/needed change of strategy	1/2	0/1	1/1	
PHLF^f^	1 B 1 C	1 B 1 C	0	

^a^FLR future liver remnant

^b^standardized FLR

^c^DH degree of hypertrophy

^d^kinetic growth rate

^e^complication classification by CIRSE [5]

^f^PHLF post hepatectomy liver failure [16]

## Discussion

This study shows that femoral access for hepatic vein embolization during DVE is feasible with a high rate of technical success, including in patients where embolization of multiple veins is considered necessary. The benefit of embolizing liver veins from the vena cava is that it enables access to cranial and medial branches that can be difficult to reach from a transhepatic access. Also, in theory, there is a risk of bleeding following transhepatic access, although we rarely see bleeding as a complication following regular PVE since the puncture tract always is embolized. As earlier described, we experienced some challenges using transhepatic access in a patient where a cranial vein branch could not be accessed. The patient later needed re-embolization of both portal and hepatic vein branches (transfemorally) due to insufficient growth of the FLR. We also experienced one case of embolizing the middle hepatic vein rather than the right hepatic vein. This was one of the early cases in the cohort, where a distal branch from the middle hepatic vein, draining the anterior lower right segment (segment 5), was accessed instead of the right hepatic vein. This was not acknowledged during the procedure, but on the post DVE CT. This has also been described earlier in cases with a transjugular access [4, 10].

**Fig. 2:**
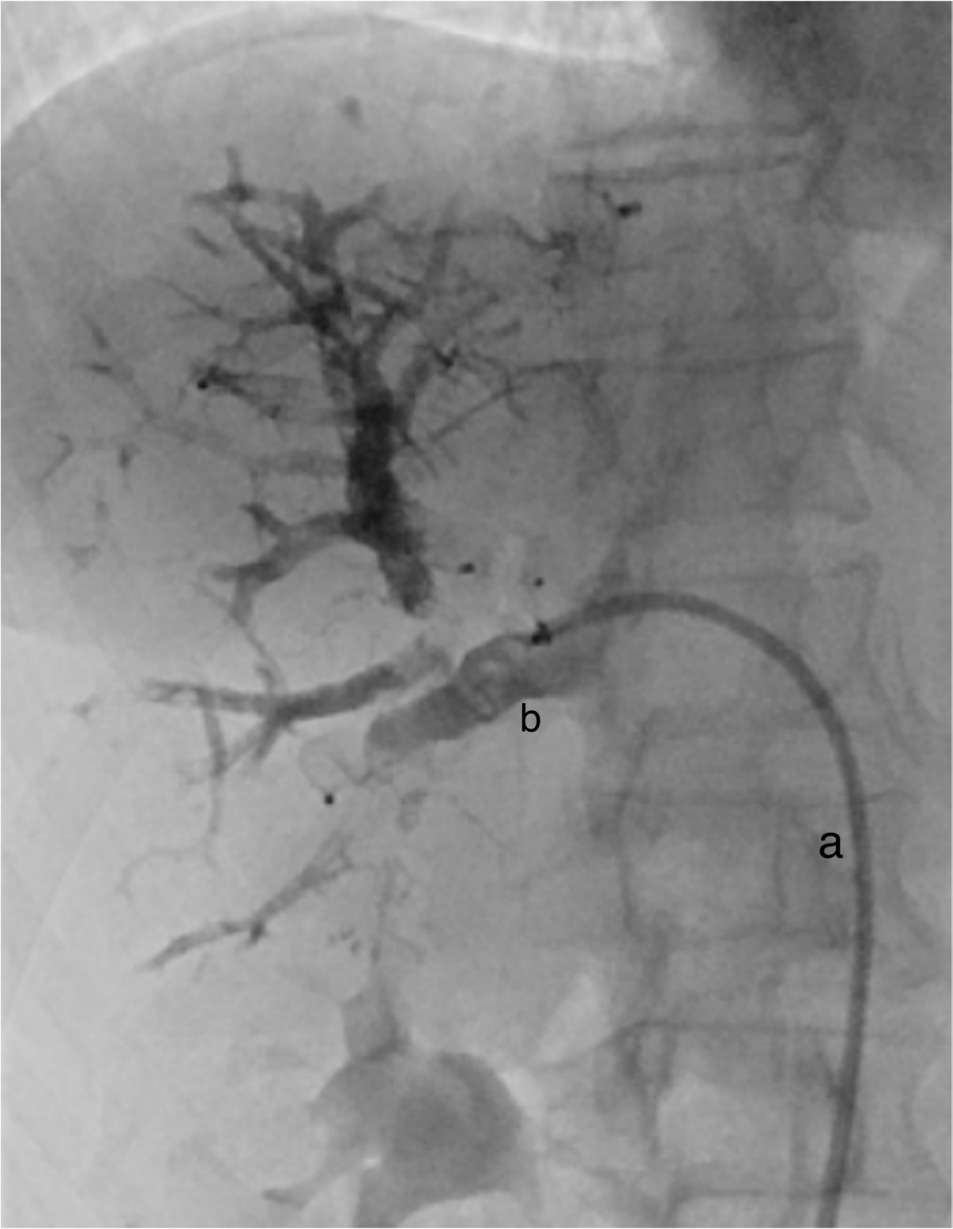
Accessory right hepatic vein embolization using a vascular plug from transfemoral access. Fluoroscopy screen save image. A vascular sheath (**a**) is placed in the accessory right vein, and a vascular plug (**b**) is placed with > 1 cm distance from the inlet to the inferior vena cava

The volumetric outcome of the DVE procedure is likely not influenced by the access per se, as long as a sufficient embolization is achieved. Anatomical suitability should be the main reason to choose the route of access [3]. In cases where the anatomy of the right hepatic vein includes only a few early branches, a transhepatic access would be appropriate, as a single plug might be enough. However, in cases with several smaller vein branches and a steep angle at the hepato-caval junction a transjugular access seems more pragmatic. In cases with a less steep angle, a transfemoral approach is a feasible option. We find the transfemoral access particular practical in cases with an accessory right hepatic vein, often with a relatively caudal inlet to the vena cava, Fig. 2. In a complex procedure such as DVE the logistics in the angiosuite is of importance. We find the femoral access easier in terms of logistics in the angio suite compared to the transjugular approach, and therefore it is our preferred access in DVE in non-transhepatic cases. The just recently published study on transfemoral vs transjugular access by Steffen et al. [20] showed that the time of the procedure was significantly shorter using a transfemoral access.

In the study by Steffen et al., the baseline sFLR% was higher than in our cohort, with 32.8% in the transfemoral group and 41.7% in the transjugular group, while we have used 20% as a cutoff for DVE. Five plugs were used for embolization and all but two patients reached resection. The hypertrophy rate was higher in the transfemoral group, however this finding might be linked to other confounding factors rather than the accesss itself [20]. A recent retrospective study compared transhepatic and transjugular approaches, without finding any differences in outcomes [4]. The baseline FLR volume in that study also was relatively higher compared to our cohort, and the majority of cases were completed by a single vein embolization. In cases where an extended right resection is needed, embolization of both right and middle hepatic is advocated for [8]. Apart from this, there is insufficient data to conclude regarding the exact number of veins that need to be embolized. In patients with very small FLRs (< 20%) one would expect that a meticulous embolization is more important than in borderline resectable patients. We show here that this is feasible doing from a transfemoral access. Results from multi-institutional prospective studies such as Dragon 1 [12] and the aforementioned RCTS might give answers regarding these issues.

Limitations of this study include the small cohort and retrospective assessment of outcomes. The choice for access was based on anatomical suitability which introduces a selection bias in the cohort. It would have been interesting to examine potential differences in radiation doses and fluoroscopy time. However these data were, due to a change of radiological information systems during the study period, missing for a large proportion of the patients.

## Conclusion

In conclusion, transfemoral access for hepatic embolization in DVE is a feasible option with high rate of technical success, also in patients with very small FLRs needing embolization of multiple veins.

## Data Availability

The datasets used and/or analyzed during the current study are available from the corresponding author on reasonable request.
